# Quantification of protein mobility and associated reshuffling of cytoplasm during chemical fixation

**DOI:** 10.1038/s41598-018-36112-w

**Published:** 2018-12-10

**Authors:** Jan Huebinger, Jessica Spindler, Kristin J. Holl, Björn Koos

**Affiliations:** Department of Systemic Cell Biology, Max Planck Institute of Molecular Physiology, Otto-Hahn-Str.11, 44227 Dortmund, Germany

## Abstract

To understand cellular functionalities, it is essential to unravel spatio-temporal patterns of molecular distributions and interactions within living cells. The technological progress in fluorescence microscopy now allows in principle to measure these patterns with sufficient spatial resolution. However, high resolution imaging comes with long acquisition times and high phototoxicity. Therefore, physiological live cell imaging is often unfeasible and chemical fixation is employed. Yet, fixation methods have not been rigorously investigated, in terms of pattern preservation, at the resolution at which cells can now be imaged. A key parameter for this is the time required until fixation is complete. During this time, cells are under unphysiological conditions and patterns decay. We demonstrate here that formaldehyde fixation takes more than one hour for cytosolic proteins in cultured cells. Other small aldehydes, glyoxal and acrolein, did not perform better. Associated with this, we found a distinct displacement of proteins and lipids, including their loss from cells. Fixations using glutaraldehyde were faster than four minutes and retained most cytoplasmic proteins. Surprisingly, autofluorescence produced by glutaraldehyde was almost completely absent with supplementary addition of formaldehyde without compromising fixation speed. These findings indicate, which cellular processes can actually be reliably imaged after a certain chemical fixation.

## Introduction

Fluorescence microscopy has advanced to allow for the precise localisation of individual molecules in cultured cells down to nanometer precision^1,2^. Furthermore, it is now possible to spatially resolve molecular reactions quantitatively by microspectroscopy or antibody based methods^3,4^. In principle, this allows for extraction of invaluable information about cellular functionalities, which are encoded in spatial organisation. However, sample preparation methods have not yet been co-developed to fully exploit the potential of these methods. Undoubtedly, sample preparation has to preserve the cellular state with at least the precision of the microscopic readout, in order to avoid artefacts.

Fluorescence microscopy can in principle be performed on living cells. This is optimal to observe cellular dynamics in all cases, where image acquisition is much faster than the process under investigation. However, more sophisticated superresolution and microspectroscopy methods usually require too long acquisition times to image the rapid processes in living cells^5^ and furthermore they are too phototoxic^6^. Therefore, cells have to be fixed before imaging. It is possible to cryo-fix cells in a close to physiological state for high resolution imaging^5,7–9^. However, this requires specialised equipment and knowledge and is therefore far from being standard procedure. Consequentially, cells are usually chemically fixed before high-resolution or functional imaging. The methods for chemical fixation have been developed decades ago and their impact on the structure of cells has been studied extensively by transmission electron microscopy^8,10^. Out of the methods used for electron microscopy, crosslinking by aldehydes as well as immersion in organic solvents have been adapted to fix cells for fluorescence microscopy. Aldehydes are the most widely used chemical fixatives for fluorescence microscopy, since fixation by immersing cells in organic solvents (e.g. acetone, ethanol or methanol), has been shown to denature and coagulate or extract cellular molecules and hence lead to more severe rearrangements in the cytoplasm^10–12^. The effects of aldehyde fixatives have been analysed by endpoint analysis of fixed cells by electron microscopy mainly of tissues, with the conclusion that formaldehyde (FA) penetrates these tissues faster and glutaraldehyde (GA) fixes them more permanently^10,13,14^. For electron microscopy of isolated cells, GA concentrations >1% are usually needed for an efficient fixation^15^. Such high GA concentrations are usually not used for fluorescence microscopy, because of the autofluorescence caused by GA^16^. However, cellular transmission microscopy provides mainly structural information about lipid-bilayer enclosed organelles and macromolecular complexes, while single molecules are usually not detectable. Fluorescence microscopy yields complementary information. Distribution of molecules or even their interactions can be mapped within a cell, whereas the surrounding structure of the cell remains invisible. While immunofluorescence has been used for decades to assign molecular localisation to certain cellular organelles, the last 20–30 years have seen an enormous improvement of fluorescence microscopy techniques. Yet, the possibilities to image single fluorescent molecules, quantify distributions of molecules and map their interactions within cells^1–4^, also raises the requirements for fixation methods substantially. Obviously, any changes introduced to the cell through fixation will ultimately lead to an incorrect representation of the living cell. It is therefore crucial to know, if and how molecules are rearranged upon chemical fixation. By comparing live cell imaging with cells after fixation some large-scale rearrangements may be detected and certain fixation protocols may thus be identified as inappropriate (e.g.^12,17,18^). However, fixation is necessary exactly in those cases, where artefact-free live-cell imaging is not possible. This prohibits this kind of comparison for high resolution imaging. Yet, the duration of chemical fixation can be informative here. This duration is critical since cells are in a non-physiological, partially-fixed state until fixation is completed. A change in parameters can alter cellular reactions during this phase. For a reaction in thermodynamic equilibrium, one can try to keep the reaction parameters (such as pH, temperature, pressure) constant, to maintain balance. Unfortunately, cells usually maintain their spatial organisation out of equilibrium by permanent energy consumption^19,20^. This results in spatial patterns which depend on various reaction rates and the movement of molecules involved (see e.g.^21,22^). It cannot be expected that all of the reaction rates and movements are slowed down during fixation in a balanced way and physiological patterns are therefore most likely not maintained. Thus, fixation needs to be fast in comparison to the decay of the patterns, which is ultimately limited by the molecular movements involved. However, little is known about the time necessary to chemically fix those cellular molecules (i.e. stop their motion), which could now be theoretically analysed with high precision by fluorescence microscopy. In the only systematic investigation on molecular species, fixation of membrane molecules was measured^23^. The authors concluded that not all membrane molecules can be fixed by chemical fixatives and that fixation time is at least 30 min. This is further supported by the finding that membrane molecules can be clustered, extracted or re-localised even after fixation by FA^5,12,18,24^. These results preclude the analysis of lateral distributions of membrane molecules after fixation with FA. However, membrane molecules are a special case. Fixatives need to cross the hydrophobic membranes before they are able to react with intracellular proteins. However, membrane proteins are embedded in their hydrophobic environment, where hydrophilic fixatives are considered not to be able to efficiently react with them. It is therefore not clear to what extent these results are also informative for non-membrane molecules.

Here, we measured fixation times directly on cytosolic proteins by consecutive bleaching of the fluorescent protein mCitrine in the cytoplasm of HeLa cells during the process of aldehyde-fixation. We found that fixation times greatly vary from less than 4 to more than 60 minutes, depending on the aldehydes used. We further observed blebbing of plasma membrane lipids following all tested aldehyde fixatives. Using most of the tested aldehydes, this was associated with loss of unfixed cytosolic protein from these blebs upon their disintegration. The loss of protein was not observable upon fixation with glutaraldehyde, which was also the fastest fixative. Unexpectedly, addition of formaldehyde repressed glutaraldehyde-induced autofluorescence almost completely, when added to 1-2% glutaraldehyde, without significantly compromising fixation speed or retention of cytosolic proteins. It seems therefore optimal to fix cells with at least 1% glutaraldehyde in combination with formaldehyde. The speed of the molecules involved should however always be considered, given the fact that fixation times in the order of minutes are not short when compared to most cellular processes.

These results should give a good basis for researchers to judge, if and which chemical fixative should be utilized to image a certain cellular process.

## Results

**Figure 1:**
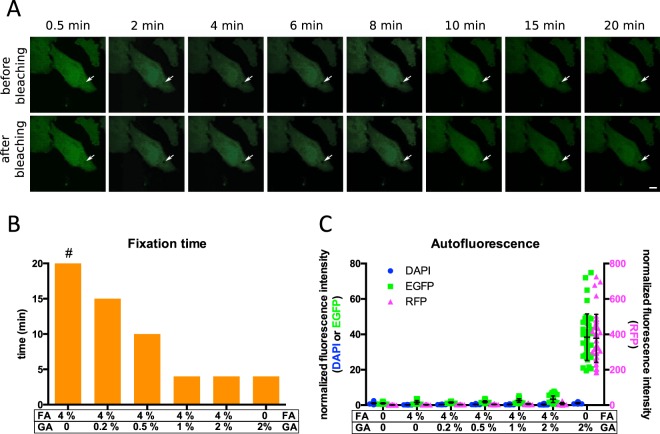
Fixation time of cytoplasmic protein and development of autofluorescence by aldehyde-fixation in HeLa cells. (**A**) Shown is a representative FRAP experiment during fixation with 2% glutaraldehyde (GA). A circular area with 2 μm diameter (white arrow) was bleached in a HeLa cell, which expressed cytosolic mCitrine, at the indicated time points after changing the medium to 2% GA. Bleaching was achieved by scanning the corresponding area with 5 laser lines of a white light laser at 100% transmission and a 405-nm laser diode at 100% intensity. The upper row shows fluorescent micrographs directly before each bleaching. The lower row shows images directly after bleaching. Scale Bar: 10 μm; (**B**) Fixation time for cultured HeLa cells as determined by consecutive bleaching and fluorescence recovery of mCitrine during chemical fixation using the indicated aldehyde concentrations. Depicted are the time points after which no further diffusion of mCitrine was observed except for 4% FA, 0% GA (**#)**, where diffusion was measurable during the whole course of the 20-min experiment after fixation and also in separate experiments after more than 60 min (Figs S1 and S2). (**C**) Increase in autofluorescence of HeLa cells upon fixation with the indicated concentrations of aldehydes in 3 different fluorescence channels corresponding to blue (DAPI; excitation (ex.) 360–370 nm/emission (em.) 420–470 nm), green (EGFP; ex. 460–480 nm/em. 495–540 nm) and red (RFP; ex. 535–550 nm/em. 570–625 nm) fluorescence measured by widefield microscopy normalised to living cells. Data is shown for single cells (coloured symbols) and mean (black lines). All autofluorescence measurements in EGFP and RFP channels are significantly higher (p < 0.001 using student's t-test) than in living cells. All measurements in DAPI channel, except after fixation with 2% GA alone (n.s.), are significantly lower (p < 0.001 using student´s t-test) than in living cells. n = 5 independent experiments (40–83 cells).

To measure the time after application of a chemical fixative until movement of cytoplasmic proteins is halted, we performed fluorescence recovery after photobleaching (FRAP) experiments. For this, HeLa cells were transfected with the cytoplasmic fluorescent protein mCitrine. A circular area with 2 μm diameter within these cells was then repeatedly bleached at defined time points after the onset of fixation (Fig. 1A). Fluorescence recovery of freely diffusing cytosolic protein was too fast to be measured in our confocal microscope. However, if the protein is fixed, it does not diffuse into the bleached area. An increase in fluorescence intensity in the spot after bleaching is thus a measure for diffusion of unfixed cytosolic mCitrine back into the bleached area. However, dark state recovery of the fluorophore can also contribute to the observed fluorescence recovery^25^. To control for the latter, we performed the same bleaching protocol with cells that were fixed for 1 h (Fig. S1). If the fluorescence recovery does not differ from this control, it can be concluded that no more fixation took place within the following hour. The first time point for which this is the case can therefore be considered as the time when fixation was completed. Using this method, we found that fixation with 4% FA, which is the standard fixative for fluorescence light microscopy, takes more than 20 min (Figs 1B and S1). Radial analysis of full recovery curves obtained after more than 1 h showed some residual diffusion of mCitrine, indicating that even the controls showed not only dark state recovery in this case (Fig. S2). During the first 20 min of exposure to 3% of the small aldehyde glyoxal in phosphate buffered saline, diffusion closed the bleached spot before the first post-bleaching image could be acquired, indicative of no fixation of cytoplasmic protein within this time. After more than 60 min the onset of fixation was measurable (Fig. S3). In contrast, 2% GA, which is often used as a fixative for electron microscopy, fixed cytoplasmic proteins within 4 min (Figs 1B and S1). In this case, no residual movement was measurable in the controls that were fixed for 1 h, demonstrating that the controls show only fluorescence dark state recovery in this case (Fig. S2). GA is not routinely used for fluorescence light microscopy, since it is notorious to cause high autofluorescence^16^. Quantification of autofluorescence showed a near 400-fold increase in red fluorescence (excitation (ex.) 535–550 nm/emission (em.) 570–625 nm), a 40-fold increase in green fluorescence (ex. 460–480 nm/em. 495–540 nm), but no increase in blue fluorescence (ex. 360–370 nm/em. 420–470 nm) (Fig. 1C). This autofluorescence could be quenched completely in the green and to approximately 5-fold in the red fluorescence channels respectively by application of first 100 mM NH_4_Cl for 40 min and subsequently 5 mg/mL NaBH_4_ for 2 h (Fig. S4). However, this procedure is relatively time-consuming, and its influence on the structure of cells is largely unknown. Interestingly, fixation by a combination of GA and FA (but not by a consecutive addition of the two (Fig. S4)) results in much lower autofluorescence than GA alone, even if the same concentration of GA is used (Fig. 1C). Combinations of 4% FA with GA at 1-2% yielded in fixation times of <4 min, similar to 2% GA alone (Fig. 1B).

**Figure 2:**
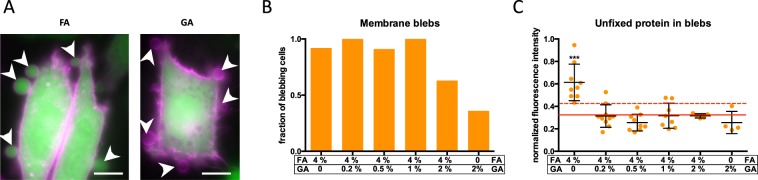
Membrane blebbing and associated loss of cytosolic protein in HeLa cells upon fixation with glutaraldehyde (GA) and formaldehyde (FA). (**A**) Shown are representative fluorescence microscopy images of HeLa cells transfected with cytoplasmic EGFP (green) and stained with the membrane marker DiIC12 (magenta) 9.5 min after application of 4% formaldehyde (FA) and 9 min after application of 2% glutaraldehyde (GA). See Figs S5 and S7 for complete time series. White arrowheads point to plasma membrane blebs that develop during fixation and are filled with (FA) or devoid of (GA) cytoplasmic EGFP. Scale bar: 10 μm; (**B**) Depicted is the fraction of HeLa cells that showed membrane blebs after fixation with the indicated concentrations of FA and GA as identified by DiIC12-staining (compare Figs S5–S7). n = 6 experiments (8–12 cells). (**C**) Depicted is the mean fluorescence intensity of cytoplasmic EGFP in the plasma membrane blebs normalised to the mean fluorescence intensity of the corresponding cell body for each blebbing cell (orange circles) and the mean +/− s.d. (black lines). The solid red line and the dashed red line indicates mean and the mean + s.d. of the background fluorescence intensity around the cells. ***p < 0.001 vs background fluorescence intensity using student´s t-test; n = 6 experiments (4–11 blebbing cells).

Lipids are not fixed at all using aldehyde fixatives^23^. In agreement with this, fluorescent microscopy of cells stained with the lipid dye DiIC12 showed extensive plasma membrane blebbing during the fixation process as visualised by fluorescence microscopy (Figs 2A,B and S5–S7). These blebs disappeared between two frames either spontaneously or, at the latest, after treatment with the detergent triton X-100, which is regularly used to permeabilise cells for antibody staining^26^. This is indicative of a bursting of these blebs, since dead fixed cells should not be able to retract blebs, which is an active energy-driven process^27^. Furthermore, retraction of blebs is a process, which happens on the timescale of minutes and should therefore be resolvable in our time series microscopy. Yet, even time series with 10 s time-resolution did not show any retracting blebs. Bleb formation in cultured cells upon FA fixation is in agreement with the literature^28^. We observed such blebs also during fixation by GA, glyoxal and acrolein (Figs 2A,B and S5–7). During FA, glyoxal or acrolein fixation, these blebs were filled with cytoplasmic protein, which eventually got lost upon disintegration of the blebs (Figs 2A,C, S3, S5 and S6). Consequentially, fixation with FA, glyoxal or acrolein induced a loss of unfixed cytoplasmic protein. Upon fixation that included GA, blebs were free of cytoplasmic protein, indicating that no loss of cytoplasmic protein arose due to this process (Figs 2A,C and S7). However, fluorescence intensity of EGFP decreased to 30 +/− 3% (mean +/− s.d.; Fig. S8A) within 1 min upon application of 4% FA + 1% GA. This reduction in fluorescence could in principle be due to interaction of the aldehydes with the fluorescent proteins or to loss of the protein from the cell. This loss of fluorescence was also measurable with 4% FA alone (to 30 +/− 10%) and to a lesser extent with 2% GA alone (38 +/− 4%; p < 0.0001; Fig. S8B). This indicates that the extent of this rapid fluorescence loss depends on the type of aldehyde used. It further depends on the fluorescence protein used, since the loss of fluorescence upon addition of 4% FA and 1% GA was significantly lower for cytosolic mCherry (46 +/− 6%; p < 0.0001; Fig. S8A). To get additional insight into the permeablization of the plasma membrane during fixation with GA, we have measured membrane integrity by incubating cells with the fluorescent dye propidium iodide (PI) upon fixation with FA + GA and GA-only. This small dye (0.668 kDa) can only enter cells with impaired plasma membrane integrity. Within the first two minutes of fixation there was no significant uptake of the dye in any of the cases (Fig. S9). This indicates that the membrane also remains impermeable during this time to proteins, which are approximately two orders of magnitude bigger (e.g. EGFP: 27 kDa). Therefore, the initial loss of fluorescence is likely due to interactions of the aldehydes with the fluorescent protein. Using 4% FA in combination with 1% GA, fluorescence intensities remained thereafter stable over the first 20 min, which is the time frame during which blebbing was observed. However, when 4% FA was used as a fixative, we observed a consistent drop of fluorescence to 71 +/−13% between 1 and 20 min (Fig. S8B; p < 0.001). This further supports the finding that there is significant loss of cytoplasmic material, when 4% FA is used and that this can be diminished by the addition of 1% GA. In addition, a membrane-bound fluorescent protein (mCherry attached to the plasma membrane by the membrane-anchor of kRas; see methods section) was detected in the artificial blebs that occurred during FA-fixation, but not in those that occurred during GA-fixation (Fig. S10). These results are in agreement and complement previous work that found lateral movement, re-localisation and extraction of membrane proteins after FA-fixation^12,18,23,24^.

The fixation of cytoplasmic material preceded the permeabilization of the cells in all cases, where GA was added. However, this effect was the most pronounced when 2% GA alone was used. In this case, there was a very slow uptake of PI within the first 20 min of fixation (Fig. S9). Yet, cytoplasmic protein was completely fixed after <4 min (Fig. 1B) and the influence of GA on the fluorescence of cytoplasmic EGFP was maximal after 1 min (Fig. S8), indicating that GA can traverse the membrane without permeabilizing it. Interestingly, when 4% FA in combination with 1-2% GA was used as fixative, permeabilization of cells occurred as a stochastic process over a relatively broad time from 2–20 min. With higher GA content, cells tended to get permeabilized later (Fig. S9).

Temperature and pH are important reactions parameters. If they are altered during the fixation process, it can be expected that even those physiological reactions that are in thermodynamic equilibrium are changed before fixation is completed. Therefore, all previous measurements in this study were performed by fixing the cells at physiological temperature and pH. However, FA-fixation at 4 °C or on ice is quite frequently performed. We therefore performed the same measurements as above on HeLa cells during FA-fixation at 4 °C. Similar to FA-fixation at 37 °C, cytoplasmic mCitrine was not completely fixed during the first 20 min of fixation. However, we observed blebs only on 20% of the cells. The lower occurrence of blebbing might be due to a phase change in the membrane. This indicates that by lowering the temperature the loss of cytoplasmic protein might be reduced. Yet, since the fixation-time was not faster, the internal state of the cytoplasm is not better preserved. We further tested a recently published protocol utilising glyoxal in combination with ethanol at pH 4^29^. In our hands, fixing cells with this protocol at 37 °C led to blebbing in all observed cells accompanied by a complete loss of fluorescent, cytosolic protein (mCherry). Further, we observed a redistribution of plasma membrane bound protein (mCherry-tkRas) and lipid (DiIC12) to intracellular compartments (Fig. S11).

**Figure 3:**
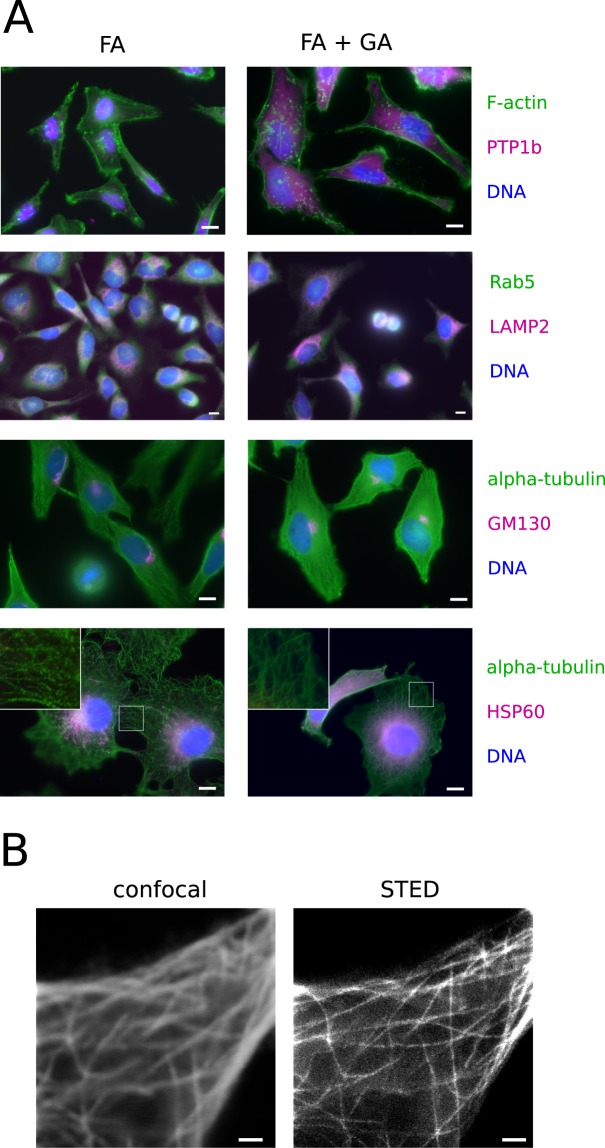
Different fluorescent stainings in cells fixed with formaldehyde (FA) and a combination of glutaraldehyde (GA) and FA. (**A**) HeLa cells (rows 1–3) or Cos7 cells (row 4) have been fixed with 4% formaldehyde (FA; left column) or 1% GA in 4% FA (FA + GA; right column). The cells have been permeabilised and the molecules indicated on the right of each row in the corresponding colours have been fluorescently labelled. The proteins PTP1b, Rab5, LAMP2, alpha-tubulin and HSP60 have been labelled by indirect immunofluorescence (more antibody stainings are presented in Fig. S12). F-actin has been labelled by Alexa488 coupled to Phalloidin. The DNA has been labelled with the intercalating dye Hoechst 33342. All experiments have been repeated three times and the images shown are representative. Antibody dilutions have been freshly prepared for each repetition and experiments after both fixations have been performed in parallel with the same antibody dilutions. The inserts in the last row show magnifications (3.3x) of the boxed area in the corresponding images. Scale bars: 10 μm (**B**) Representative images of alpha-tubulin stained by indirect immunofluorescence with the fluorescent dye Alexa-647 in HeLa cells imaged by confocal (left image) and stimulated emission depletion (STED) microscopy (right image) after fixation with 1% GA in 4% FA. Scale bars: 1 μm.

The previous results suggest fixation with 1% GA in 4% FA at 37 °C and physiological pH is the most favourable approach to achieve the best fixation speed in combination with little autofluorescence. They also show that the fluorescence from expressed proteins (mCitrine, EGFP, mCherry) or lipid dyes (DiIC12) is preserved following this treatment. However, for antibody-based methods, epitope accessibility is also a crucial factor. We therefore compared fixation in 1% GA plus 4% FA with the standard fixative, i.e. 4% FA. In diffraction-limited immunofluorescence experiments, we compared 19 different stainings of cellular compartments (17 antibody-stainings, phalloidin-alexa-488 and Hoechst 33342). The stainings with phalloidin and Hoechst 33342 worked equally well after both fixation methods. 14 out of the 16 antibody stainings, which have been previously established and used in FA-fixed samples in our lab, also localised to the same cellular structures (Figs 3 and S12). Only stainings with antibodies against HSP60 and cFos did not show the expected mitochondrial and nuclear localisations after fixation with 1% GA and 4% FA (Figs 3 and S12). On the other hand, some stainings showed more signal after fixation in 1% GA and 4% FA. For example, using the antibody against alpha-tubulin the microtubules were completely decorated with fluorophores. After fixation with 4% FA, only dotted stainings along the microtubules were detectable, when the same antibody concentrations were used in parallel experiments (Fig. 3). This was further confirmed by superresolution (stimulated emission depletion (STED)) microscopy (Figs 3 and S13). It has been shown before, by detailed analysis using single molecule localization microscopy, that microtubules can be fixed by 3.7% FA, without major structural artefacts^30^. Using a 4% FA and 1% GA, we did also not observe obvious structural artefacts such as breakage in microtubules. Given the rapid dynamics of microtubule polymerisation and de-polymerisation, it remains however unclear, how much the living state is represented in detail. Further, testing of an antibody against PTP1b, which we had previously not validated for immunofluorescence, gave significant fluorescence signal only after fixation with 1% GA in 4% FA (Fig. 3). The localisation of this ER-bound protein throughout the cell was in agreement with its localisation in living cells^31^. These results thus show that there is no general sterical hindrance due to more efficient crosslinking after fixation with 1% GA in 4% FA. However, epitope accessibility is specific for combinations of antibody and fixation methods and none of these two fixatives are generally favourable in this regard.

## Discussion

The presented results provide an overview on fixation times, autofluorescence, membrane blebbing and loss of cytoplasm during different aldehyde fixations. We have mainly focused on the aldehydes which are the most widely used fixatives, FA and GA, and their combinations. Other small aldehydes, acrolein and glyoxal, which are only rarely used for fixation, gave inferior results to FA and GA under the tested conditions. Generally, our results suggest to perform chemical fixation for cytoplasmic proteins using a combination of FA and GA with GA-concentrations of at least 1% at physiological temperature and pH. This fixation method was among the fastest, retaining most of the cytosolic proteins, while producing only very minor autofluorescence. This autofluorescence may be quenched even further for very sensitive measurements. For membrane associated proteins, this fixation method seems to be also beneficial, since these proteins were not redistributed to fixation-induced plasma membrane blebs. Even though addition of GA is also clearly beneficial for structural preservation^32^, combinations of GA and FA are rarely used for modern fluorescence light microscopy. In these applications, usually very low concentrations (often around 0.1%) of GA are used to prevent autofluorescence. However, we show here that GA concentrations need to be at least 1% to achieve optimal fixation times. Surprisingly, our results also showed that the autofluorescence induced by GA is very much reduced in the presence of FA, which allows for higher concentrations of GA and thereby much faster fixation.

The exact mechanism of autofluorescence-formation by the binding of GA to proteins is not clear. It has been proposed however, that the incorporation of several GA molecules at one binding site is necessary to form fluorophores^16^. It is therefore possible that incorporation of FA molecules instead of one or more of the GA molecules prohibits the formation of these fluorophores. This mechanism would be also in agreement with our observation, that application of FA after GA-fixation did not reduce GA-induced autofluorescence.

The speed of fixation depends primarily on two factors: The diffusion of the fixative into the cell and the speed of the crosslinking reaction. Diffusion speed has been shown to be a critical factor for the fixation of organs or relatively thick tissues^14^. GA diffuses significantly slower into these tissues than FA^10,13^. Using the formula developed for the diffusion of fixatives in tissues^14^ and the diffusion values for GA (k ≈ 0.5; e ≈ 2^10^), one would estimate for a monolayer of adherent cells (thickness D ≈ 0.005 mm^33^) a diffusion time of T = kD^e^ = 0.5 × 0.005^2^ = 0.0000125 h. Compared to the fixation times presented in this manuscript and those of membrane molecules^23^, this diffusion time is actually negligible in such thin samples. It becomes however limiting in thicker tissues, where diffusion times are often in the order of hours^10,13,14^.

Our results confirm that diffusion of both fixatives into the cells happens within <1 min, since both FA and GA led to a significant decrease in fluorescence during this time. Both FA and GA were able to penetrate the cell without immediately compromising the integrity of the plasma membrane. Subsequently, cytoplasmic protein got fixed after <4 min when 1-2% GA were used followed by a permeabilization of the membrane at later time points. In contrast FA crosslinked the cytoplasmic proteins slower and led to a faster permeabilization of cells. The results thus indicate that in cultured cells fixation times are limited by the time it takes to crosslink proteins and that the faster fixation time of glutaraldehyde is due to its higher crosslinking capabilities^28,34^.

Our results further confirmed that epitope accessibility depends on the combination of antibody and fixation^26^. However, most antibodies that are established in FA-fixed samples worked also after fixation with a combination of FA and GA. This demonstrates that there is no general sterical hindrance for antibodies due to better crosslinking by GA. Yet, some antibodies only bind after one of the fixation methods. This is thus likely due to specific epitope coverage^26^ and should be solvable by using antibodies for different epitopes on the same protein. We think that an appropriate fixative, which preserves the cell in a closer to physiological state, should usually be the primary objective. The antibody should comply with the fixation method not the reverse, whenever possible. However, this also depends strongly on each particular experiment.

Based on the measured process and the desired readout, different fixation methods may be sufficient or even slightly beneficial. Due to the wide variety of fluorescence light microscopy measurements this needs to be assessed on a case-by-case basis. This however necessitates knowledge about the course of fixation. The changing of pH or temperature will change the physiological state of the cell in our experiments did not result in faster or more efficient fixation. It may therefore only be advisable to do so in very special cases. One such example could be that fixation with 4% FA on ice (but not at 37 °C) might be sufficient, if the amount of a cytoplasmic protein is to be quantified in a cell, but the subcellular distribution is not of interest. This fixation is much slower than fixation with a combination of GA and FA at 37 °C, however the autofluorescence in the green to red spectrum should be slightly lower and due to the reduced blebbing at 4 °C, loss in cytoplasmic protein is likely less than after FA-fixation at 37 °C. Further, if blue fluorophores are used, fixation with 2% GA can also be efficient, since we found aldehyde-induced autofluorescence only in the green and red part of the spectrum. To avoid artificial clustering of transmembrane receptors, addition of small amounts of GA (≤0.25%) to FA or small amounts of detergents might be sufficient^18^. Additionally, to find out if a protein of interest is located in a certain membrane-bound organelle, even the rather inefficient fixation with formaldehyde at 37 °C can be sufficient and this has actually been utilised in numerous studies over decades. Even though this is not optimal as e.g. a putative cytoplasmic fraction might be underestimated due to extraction of cytoplasmic protein.

Modern microscopy is however able to measure the distribution of cellular macromolecules and even their interaction at much higher resolution. In this context, it is important to note that even the fastest tested fixations occur in the order of minutes, which is comparatively slow relative to many cellular processes. Imaging a fixed cell can therefore never be considered as taking a snapshot of a living cell at a distinct point in time, as it is often implied. In addition to a good understanding of the process of fixation, also knowledge regarding the investigated cellular process itself is necessary to judge, if a certain fixation is actually suited for a particular measurement. Some cellular structures might be in, or close to, thermodynamic equilibrium. These are relatively uncritical to fix, since they will not change as long as the reaction parameters (e.g. temperature, pressure, pH) are not changed. Yet, they still could be altered if extraction from the cytoplasm changes concentrations of reaction partners. However, most processes in a living cell are maintained out of equilibrium by permanent energy consumption, as this is an inherent feature of life^19,20^. For all of these processes, fixation speed is very critical, since it needs to be faster than the decay of these patterns upon withdrawal of energy. A well investigated example for a spatial regulated pattern in the cytoplasm is signal transduction to the nucleus after sensing of extracellular stimuli at the plasma membrane of cells by receptor molecules. The receptors initiate a cascade of activation events in the cytoplasm, usually by the phosphorylation of proteins. These phosphorylated proteins diffuse away from the plasma membrane. However, in the cytoplasm phosphatase activity is dominating, which reverts their phosphorylation^21^. The phosphorylation of proteins at the plasma membrane (the source) and their diffusion into the dephosphorylating cytoplasm (the sink), creates a gradient of phosphorylation from the cytoplasm into the interior of the cell^21^. This gradient is dependent on at least three factors: the rates of phosphorylation and de-phosphorylation and the speed of diffusion. Diffusion will be slowed down by fixation, but also phosphorylation and de-phosphorylation are non-equilibrium processes and will be altered during the lethal fixation process^35^. Since one cannot expect these processes to be slowed in a balanced way, it will alter the appearance of the gradient in the cell after fixation. Cytosolic proteins diffuse with 10–100 μm^2^/s. Therefore, gradients in the cytoplasm could decay in the timeframe of a second or less. This can also be seen from FRAP experiments. If a spot in the cytoplasm of an unfixed cell is bleached, this represents an artificial gradient, which is not maintained by energy consumption. This spot disappears within less than a second, which can for example be seen in Fig. S3A. This is an extreme case, since it cannot be expected that upon fixation the energy is immediately depleted, but it provides a useful illustration of the fragility of diffusion-dependent patterns. Thus, it might be impossible to fix such patterns with the available chemical fixations and consequently their direct imaging in fixed cells has – to our knowledge – not yet been achieved. This is of course also true for more complicated diffusion-dependent patterns like Turing patterns, which could also be formed within cells^21^. On the other hand, structural elements such as some parts of the cytoskeleton have lower turnover rates and are therefore somewhat easier to fix.

It is of note that the fixation-artefacts that we have observed here were very obvious during the fixation process. However, extraction of lipids and proteins as well as rearrangements in their organisation would not be self-evident in an endpoint analysis of fixed cells alone. This could lead to false interpretations, if the process of fixation is not completely understood. In conclusion, the results presented here should provide a good basis for researchers to make an informed decision, if and which chemical fixation is suitable to measure a specific cellular process.

## Material and Methods

### Preparation of fixation media

A commercially available phosphate-buffered 4% formaldehyde solution was used to generate the results presented in this study (Histofix, Carl Roth GmbH, Karlsruhe, Germany). However, solutions of formalin diluted to 4% formaldehyde in phosphate buffered solution (PBS) or HEPES-buffered DMEM were also tested. Results were the same for these solutions. Glutaraldehyde (25% stock solution, Sigma-Aldrich Chemie GmbH, Munich, Germany), and acrolein (90% stock solution, Sigma-Aldrich Chemie GmbH, Munich, Germany) were diluted in PBS to the indicated concentrations. Mixtures of GA and FA were obtained by diluting GA (25% stock solution) into the commercially available phosphate-buffered 4% formaldehyde solution (Histofix). Glyoxal was either used as 3% solution in PBS or prepared as suggested in a recent publication^29^. For the latter case, 2.835 mL ddH2O, 0.739 mL ethanol, 0.313 mL glyoxal solution (40%; Sigma-Aldrich Chemie GmbH, Munich, Germany) and 0.03 mL of acetic acid were mixed and the pH was adjusted to 4 by adding NaOH.

### Preparation of cover slides for cell culture

In order to observe cells during the process of fixation without changing focus or position during addition of the fixative, a self-built flow-through chamber was used^5^. Using this flow-through chamber also ensured a controlled and smooth exchange of culture medium to fixative. It has been previously observed that aspiration of cell medium and consecutive addition of fixative lead to stronger artefacts then a direct exchange^30^. In agreement with this we found much stronger blebbing during GA-fixation, when medium was aspirated first and fixative was added subsequently. The use of this flow-chamber required the preparation of custom cover slides^36^. A 10 μm thick double-sided adhesive tape (Modulor GmbH, Berlin, Germany) was cut to the size of a 21 × 26 mm cover slide (Gerhard Menzel GmbH, Braunschweig, Germany). In the centre, a rectangular area with the dimension of the cavity of the aluminium flow chamber was cut out. The adhesive tape was glued on the cover slides (keeping the upper side of the tape covered by the release liner), sterilised in ethanol and washed with sterile H_2_O. Cover slides with adhesive tape facing upwards were placed in sterile 6-well-plates for cell culture (Sarstedt AG & Co, Nümbrecht, Germany).

### Cell Culture and Transfection

HeLa cells (ATCC No. CCL-185) and Cos-7 cells (ATCC No. CRL-1651) were cultured in Dulbecco’s Modified Eagle Medium (DMEM) supplemented with 10% fetal bovine serum (FBS), 100 µg/mL streptomycin, 100 U/mL penicillin, 1% L-Glutamine (200 mM), and 1% nonessential amino acids (all PAN-Biotech, Aidenbach, Germany). HeLa Cells were authenticated by Short Tandem Repeat (STR) analysis and did not contain DNA sequences from mouse, rat and hamster (Leibniz Institute DSMZ). Cells were regularly tested for mycoplasma infection using the MycoAlert Mycoplasma detection kit (Lonza, Basel, Switzerland) and cultured at 37 °C with 95% air and 5% CO_2_. Cells were plated on the prepared 6-well dishes containing the prepared cover slides or in glass bottom dishes (MatTek Corporation, Ashland, MA, USA) 1–3 days before the experiment. One day before imaging, HeLa cells were transfected using FuGENE^TM^ 6 (Promega, Madison, WI, USA) following the standard protocol of the manufacturer.

### Mounting of the cover slides to a temperature-controlled flow-through chamber

The assembly of the temperature controlled flow-through chamber was performed as previously described in detail^5,36^. Briefly, the cover slides with cells were removed from the 6-well plates. The release liner of the double-sided adhesive tape was removed and the cover slides were glued to the custom-made flow chamber^5^. The rectangular part in the middle without adhesive tape was mounted right under the cavity of the flow chamber. Care was taken to keep the cells moist by medium during this process. The temperature control was fixed to the flow-through chamber as previously described. Solution exchanges during cooling and warming were realised with the help of a neMESYS low-pressure syringe-pump (Cetoni GmbH, Korbußen, Germany) that enables a constant flow rate. For live-cell imaging, cells were flushed with HEPES buffered DMEM medium without phenol red and temperature was set to 37 °C. Washing the cells before fixation with PBS, as it is common practice in many fixation protocols, did not yield in significantly different results. For fixation on the microscope, the corresponding fixative was flushed with 3 μL/s and the temperature was set to 37 °C, except for one set of experiments where cells were fixed with FA at 4 °C. Temperature change was timed such that the fixative accessed the cells directly once the temperature reached 4 °C.

### Fluorescence imaging of EGFP, mCherry, mCherry-tkRas and DiIC12

HeLa cells were transfected with plasmids for EGFP only (EGFP-N1 vector, Clontech Laboratories Inc., Mountain View, CA, USA), mCherry only (this construct was obtained by replacing the sequence of EGFP by that of mCherry^37^ using classical restriction ligation method in EGFP-N1), or EGFP and mCherry-tkRas (mCherry fused to the C-terminal sequence of kRas; the last 20 amino acids of kRas anchor the membrane to the inner leaflet of the plasma membrane^38^, which has been used for plasma membrane localization of fluorescent proteins before^39^) one day before the experiment. Labelling of cells with DiIC12 was performed by incubating the cells in 2 μg/mL DiIC12 in FBS-free medium for 2 min and subsequently washing them with HEPES-buffered DMEM medium without phenol red. Cells were imaged on a widefield microscope (Olympus IX-81) with a 63 × 1.3 NA oil-immersion objective (Olympus GmbH, Hamburg, Germany). Fluorescence intensities were quantified using the software Fiji^40^.

### Fluorescence Recovery After Photobleaching (FRAP)

FRAP measurements were performed on a Leica SP5 confocal microscope using a 63 × 1.4 NA oil-immersion objective (Leica Microsystems CMS GmbH, Mannheim, Germany). Cells transfected with mCitrine (this construct was obtained by replacing the sequence of EGFP by that of mCitrine^41^ in the EGFP-N1 vector using classical restriction ligation method) were imaged by excitation with the 514-nm laser line of a white light laser at 70% power with <10% transmission with 2.65 frames/s. Bleaching was performed by scanning a circular spot of 2 μm with 5 laser lines of the white light laser (WLL) at 100% transmission (app. 1 mW per laserline) plus a 405-nm laser diode at 100% intensity. This resulted in a bleaching efficiency of >80% in fixed cells. Using less laserlines of the WLL resulted in less efficient bleaching. The additional use of the 405-nm diode reduced the amount of fluorescence recovery due to fluorescent dark states, as observed before^5^. Cells were bleached 0.5, 2, 4, 6, 8, 10, 15 and 20 min after the beginning of fixation (compare Figs 1A and S1). Fluorescence images were recorded before and after each bleaching. They were analysed using the software Fiji^40^. Background fluorescence was measured outside of the cells and subtracted from the whole image using Fiji. To correct for total bleaching, development of autofluorescence and protein loss in the cell, the mean fluorescence intensity in the bleached area (I_bleachraw_) was normalised to the mean fluorescence intensity of the whole cell (I_cell_).

$${I}_{bleach}({t}_{i})=\frac{{I}_{bleachraw}({t}_{i})}{{I}_{cell}({t}_{i})}$$

To correct for inhomogeneous distribution of fluorescence within the cell, this intensity (I_bleachspot_) was further normalised to the initial ratio between the mean in bleached area (before the first bleaching) and the mean of the whole cell.

$${I}_{norm}({t}_{i})=\frac{{I}_{bleach}({t}_{i})}{{I}_{bleach}({t}_{0})}$$

The recovery of the normalized fluorescence between consecutive time points (I_recovery_) was calculated for every time point after the first one.

$${I}_{recovery}({t}_{i})={I}_{normunbleached}({t}_{i})-{I}_{normbleached}({t}_{i-1})$$

As a control for fluorescence dark state recovery, cells that have been fixed for 1 h were bleached following the same protocol (Fig. S1). Fixation was therefore regarded as complete once the recovery of fluorescence intensity was no longer significantly higher than its control. To evaluate the level of completion of protein fixation 1 h after application of the fixative, recovery curves over the first 20 s after a single bleaching were additionally recorded. To distinguish in this case between fluorescence dark state recovery and diffusion into the bleached spot, the bleached spot was divided into three concentric rings (Fig. S2). Diffusion into the spot is faster in the outer rings, whereas fluorescence dark state recovery is equally fast in all areas of the spot^25^.

### Propidium iodide staining of cells during fixation

The cell culture medium was replaced by the fixative indicated in the corresponding Fig. S9 with 5 μM propidium iodide. After 30 min of fixation, the fixative was replaced by 20% ethanol in cell culture medium without phenol red, to permeabilize all cells. Cells were imaged on a widefield microscope (Olympus IX-81) with a 20 × 0.7 NA objective (Olympus GmbH, Hamburg, Germany). Fluorescence intensities were quantified after subtraction of the background using the software Fiji^40^.

### Immunofluorescence microscopy and stimulated emission depletion (STED) microscopy

Immunofluorescence microscopy experiments were performed on HeLa or Cos7 cells grown in glass bottom dishes (MatTek Corporation, Ashland, MA, USA). Cells were fixed for 20 min at 37 °C either in 4% formaldehyde or 1% glutaraldehyde in 4% formaldehyde.

The stainings presented in Fig. 3, were subjected to the following protocol. After washing twice with PBS, cells were permeabilised with 0.5% Triton-X100 in PBS for 5 min. After washing twice with PBS again, the samples were incubated for 60 min with the primary antibodies (see Table 1) in PBS containing 1% BSA. Labelling of Phalloidin-Alexa488 (1:200; Invitrogen Carlsbad, CA, USA) was done together with the PTP1b antibody.

**Table 1:** List of primary antibodies.

Antigen	Manufacturer	Dilution	Origin
alpha-tubulin	Abcam	1:200	rabbit
GM 130	BD Biosciences	1:200	mouse
LAMP2	Fitzgerald Industries Int.	1:50	mouse
HSP60	Sigma-Aldrich	1:100	mouse
PTP1b	Assay Biotechnology	1:200	rabbit
Rab5	Cell Signaling	1:100	rabbit
EGFR	R&D systems	1:200	goat
cFos	Cell Signaling	1:200	rabbit
pRB	Cell Signaling	1:400	rabbit
panRas	Calbiochem	1:200	mouse
Rab11	BD Biosciences	1:200	mouse
pERK	Cell Signaling	1:200	rabbit
ERK	Cell Signaling	1:100	mouse
beta-catenin	Santa Cruz	1:200	mouse
TSC2	Cell Signaling	1:200	rabbit
cJun	Cell Signaling	1:200	rabbit
MEK1/2	Cell Signaling	1:200	mouse

The antibody stainings in Fig. S12 were subjected to the following protocol: After washing twice with Tris-buffered saline (TBS), cells were permeabilised with 0.5% Triton-X100 in TBS for 5 min. After washing twice with TBS again, the samples were blocked for 30 min in Odyssey blocking buffer (Licor, Lincoln, USA) and subsequently incubated over night with the primary antibodies (see Table 1) in this buffer at 4 °C. After washing twice with TBS again, the cells were incubated with the secondary antibodies (donkey anti goat coupled to Alexa 488 for EGFR staining; chicken anti rabbit coupled to Alexa 647 for the STED imaging in Fig. 3B; goat anti mouse coupled to Alexa 546 and goat anti rabbit coupled to Alexa 488 for all other experiments; all Thermo Fisher Scientific, Waltham, MA, USA) 1:300 in Odyssey blocking buffer.

In all stainings, DNA was stained by Hoechst 33342 (250 ng/mL in PBS) for 5 min, after washing twice with PBS. Before imaging, cells were washed twice in PBS.

Widefield epifluorescence imaging was done on a Zeiss Axiovert widefield microscope using a 40 × 1.3 NA or a 63 × 1.4 NA oil immersion objective. STED microscopy was done on a dedicated STED microscope (Abberior Instruments GmbH, Göttingen, GmbH) using a 485-nm excitation laser, a 595-nm depletion laser (1 W) at 30% power and a 100 × 1.4NA oil immersion objective.

### Statistical analysis

Two-tailed, homoscedastic student’s t-tests were performed using the software Excel (Microsoft corporation, Redmond, WA, USA).

## Electronic supplementary material

Supplementary Figures 1-13

## Data Availability

The data generated for this work are available from the corresponding author upon request.
