# Supplementary material for: Quantification of protein mobility and associated reshuffling of cytoplasm during chemical fixation
Source: Sci Rep. 2018 Dec 10;8:17756. doi: 10.1038/s41598-018-36112-w (PMC6288139; doi:10.1038/s41598-018-36112-w)
Supplement: Supplementary file 1 — Supplementary Figures 1-13 [file 41598_2018_36112_MOESM1_ESM.docx]

**Quantification of protein mobility and associated reshuffling of cytoplasm during chemical fixation**

Jan Huebinger *, Jessica Spindler, Kristin J. Holl, Björn Koos

**Supplementary Information**

**Supplementary figure 1:** Mobility measurement of mCitrine in HeLa cells by FRAP during the fixation with different aldehydes

**Supplementary figure 2:** FRAP of mCitrine in HeLa cells 1 h after onset of fixation

**Supplementary figure 3:** FRAP experiments on a HeLa cell during fixation with 3% glyoxal

**Supplementary figure 4:** Autofluorescence of HeLa cells after fixation with 2% glutaraldehyde and quenching with different protocols

**Supplementary figure 5:** Fluorescence imaging of cytosolic EGFP and the membrane-marker DiIC12 in HeLa cells during fixation with 4% formaldehyde

**Supplementary figure 6:** Fluorescence imaging of cytosolic EGFP and the membrane-marker DiIC12 in HeLa cells during fixation with 5% acrolein

**Supplementary figure 7:** Fluorescence imaging of cytosolic EGFP and the membrane-marker DiIC12 in HeLa cells during fixation with 2% glutaraldehyde

**Supplementary figure 8:** Quantification of the loss of fluorescencence of cytosolic EGFP and mCherry upon fixation with 4% formaldehyde and 1% glutaraldehyde

**Supplementary figure 9:** Propidium iodide staining of HeLa cells during fixation

**Supplementary figure 10:** Fluorescence imaging of HeLa cells expressing plasma membrane associated mCherry during fixation

**Supplementary figure 11:** Fluorescence imaging of cytoplasmic mCherry, mCherry bound to the plasma membran­­e and the plasma membrane marker DiIC12 in HeLa cells during fixation with 3% glyoxal and 20% ethanol at pH 4

**Supplementary Figure 12:** Different immunofluorescence stainings in cells fixed with formaldehyde (FA) and a combination of glutaraldehyde (GA) and FA

**Supplementary Figure 13:** Confocal and STED microscopy of alpha-tubulin labelled in HeLa cells fixed with formaldehyde (FA) and a combination of glutaraldehyde (GA) and FA

**Supplementary figure 1: Mobility measurement of mCitrine in HeLa cells by FRAP during the fixation with different aldehydes**

HeLa cells were successively bleached at one circular area of 2 μm diameter as shown in figure 1A. Shown are fluorescence recoveries (fractions of the fluorescence intensities before bleaching) of mCitrine in the bleached spot during the course of fixation in the indicated fixatives (blue symbols) and in a control experiments starting 1 h after beginning of fixation (green symbols). The control measurement is necessary to correct for fluorescence recovery from dark states of the fluorophores, which are not related to the diffusion of the fluorophores. The indicated time points are the time points of recovery, bleachings took place at the previous time points. All fluorescence intensities were normalized to the total fluorescence of the cell, to correct for the amount of bleached material and normalized to the initial ratio between the bleached spot and the whole cell, to correct for inhomogeneity of fluorescence distribution among the cells. Data is shown as single cell data (colored symbols) and mean +/- s.d. of 4-8 independent (single cell) measurements. Asterisks indicate significant differences between the recovery during fixation vs the corresponding control (two-tailed student’s t-test; * p<0.05; ** p<0.01; ***p<0.001).

**Supplementary figure 2: FRAP of mCitrine in HeLa cells 1 h after onset of fixation**

Shown are fluorescence recovery curves of mCitrine in HeLa cells that have been fixed for 1 h in 4% FA (a) or 2% GA (b). The fluorescence recovery is measured in the concentric rings within the bleached spot (c). The data in (a) and (b) is shown as mean (solid colored lines) +/- s.d. (dotted colored lines) and linear fits (black dotted lines) of 13 (a) or 14 (b) cells from 3 independent (single cell) experiments. Diffusion into the spot is faster in the outer rings, whereas fluorescence dark state recovery is equally fast in all areas of the spot. The slope of the fluorescence increase is highest in the outer and lowest in the inner ring after FA-fixation (outer ring: 0.2444 s^-1^; middle ring: 0.1671 s^-1^; inner ring: 0.1208 s^-1^), indicating some residual movement. After GA-fixation, the slopes are very similar in all three rings (0.1754 s^-1^; 0.1465 s^-1^; 0.1401 s^-1^), indicating a complete fixation.

**Supplementary figure 3: FRAP experiments on a HeLa cell during fixation with 3% glyoxal**

**A**) Shown is a representative HeLa cell expressing cytosolic mCitrine during a FRAP experiment during fixation. A circular area with 2 μm diameter was bleached at the indicated time points after changing the medium to 3 % glyoxal. Bleaching was done by scanning the corresponding area with 5 laser lines of a white light laser at 100% transmission and a 405-nm laser diode at 100% intensity. The upper row shows fluorescent micrographs directly before each bleaching. The lower row shows images directly after bleaching. The shown experiment is representative for three independent (single cell) experiments. The bleached area was not identifiable in all three experiments. **B**) Shown is a FRAP series on the same cell as in A) that started after the cell has been exposed for 60 min to 3% glyoxal. In two out of three experiments, it was possible to bleach a spot in the cytoplasm. Scale Bars: 20 μm

**Supplementary figure 4: Autofluorescence of HeLa cells after fixation with 2% glutaraldehyde and quenching with different protocols**

Depicted is the autofluorescence in 3 different fluorescence channels corresponding to DAPI- (ex. 360-370 nm/ em. 420-470 nm; blue; left y-axis), GFP- (ex. 360-370 nm/ em. 420-470 nm; green; left y-axis) and RFP- (ex. 535–550 nm/ em. 570–625 nm; magenta; right y-axis) fluorescence measured by widefield microscopy. All samples have been fixed for 20 min in 2 % glutaraldehyde. Subsequently they have been left untreated, treated with 4 % FA only or treated for 40 min with 100mM NH_4_Cl and subsequently with the indicated concentrations of NaBH_4_ for the indicated times. Fluorescence was normalized to that of living cells under the same imaging conditions. Shown are single cell measurements (colored symbols) and mean +/- s.d.; ***: significantly higher (p<0.001 using student´s t-test) than living cells; ns: no significant difference to living cells; Data was obtained in 5 independent experiments (30-45 cells)


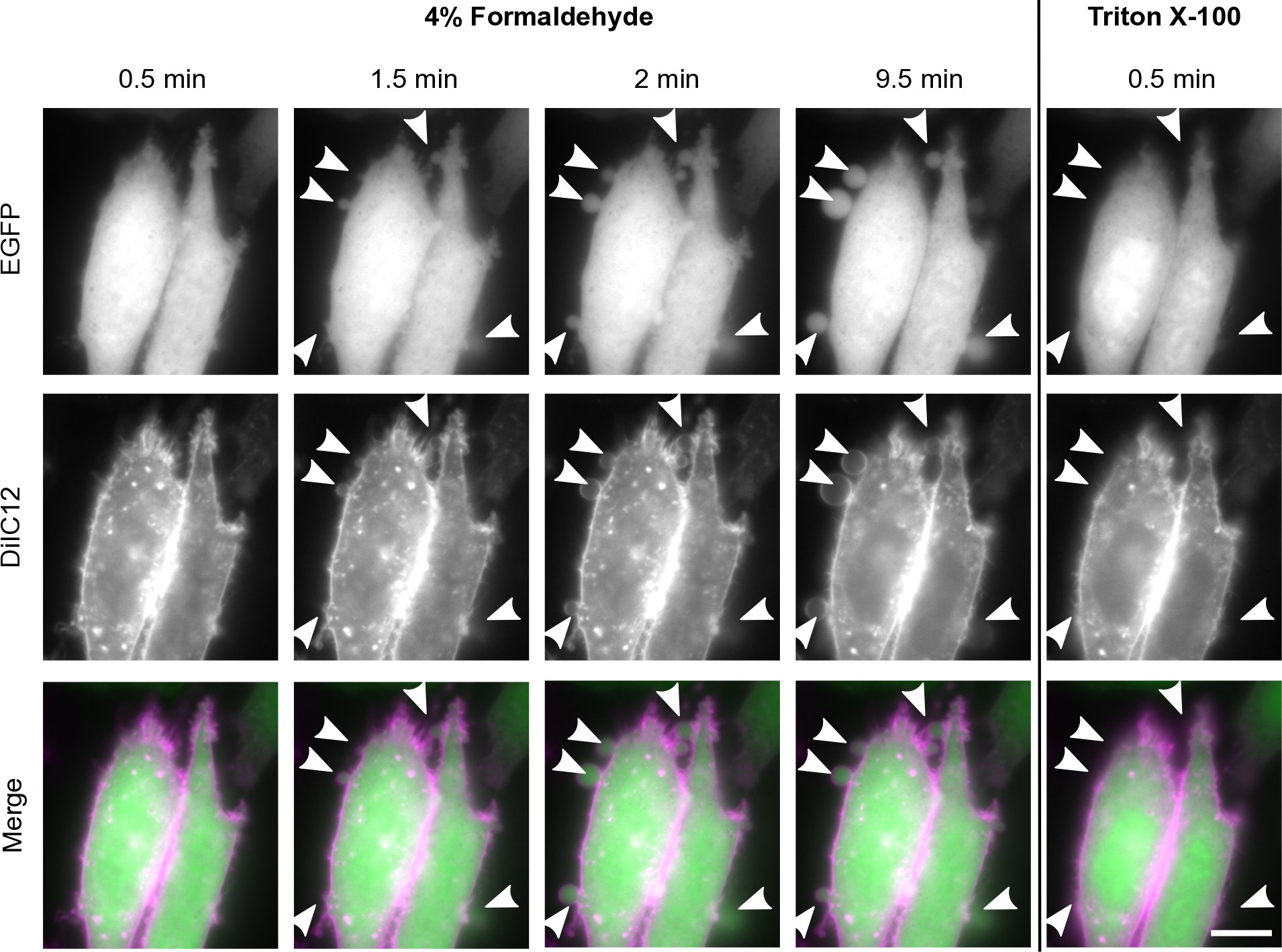


**Supplementary figure 5: Fluorescence imaging of cytosolic EGFP and the membrane-marker DiIC12 in HeLa cells during fixation with 4% formaldehyde**

Shown are representative fluorescence microscopy images of HeLa cells transfected with cytoplasmic EGFP (upper row, green in lower row) and stained with the membrane marker DiIC12 (middle row, magenta in lower row) during the course of fixation in 4% formaldehyde and subsequent permeabilization by 0.5 % Triton X-100. The contrast is maximized for each individual image to correct for the loss of fluorescence during fixation. White arrowheads point to membrane blebs filled with cytoplasmic EGFP that develop during fixation and are rapidly dissolved during permeabilization. Note also the difference in contrast upon addition of Triton X-100 between nucleus and cytoplasm (black double arrow). This experiment is representative of 6 experiments (12 cells); scale bar: 10 μm


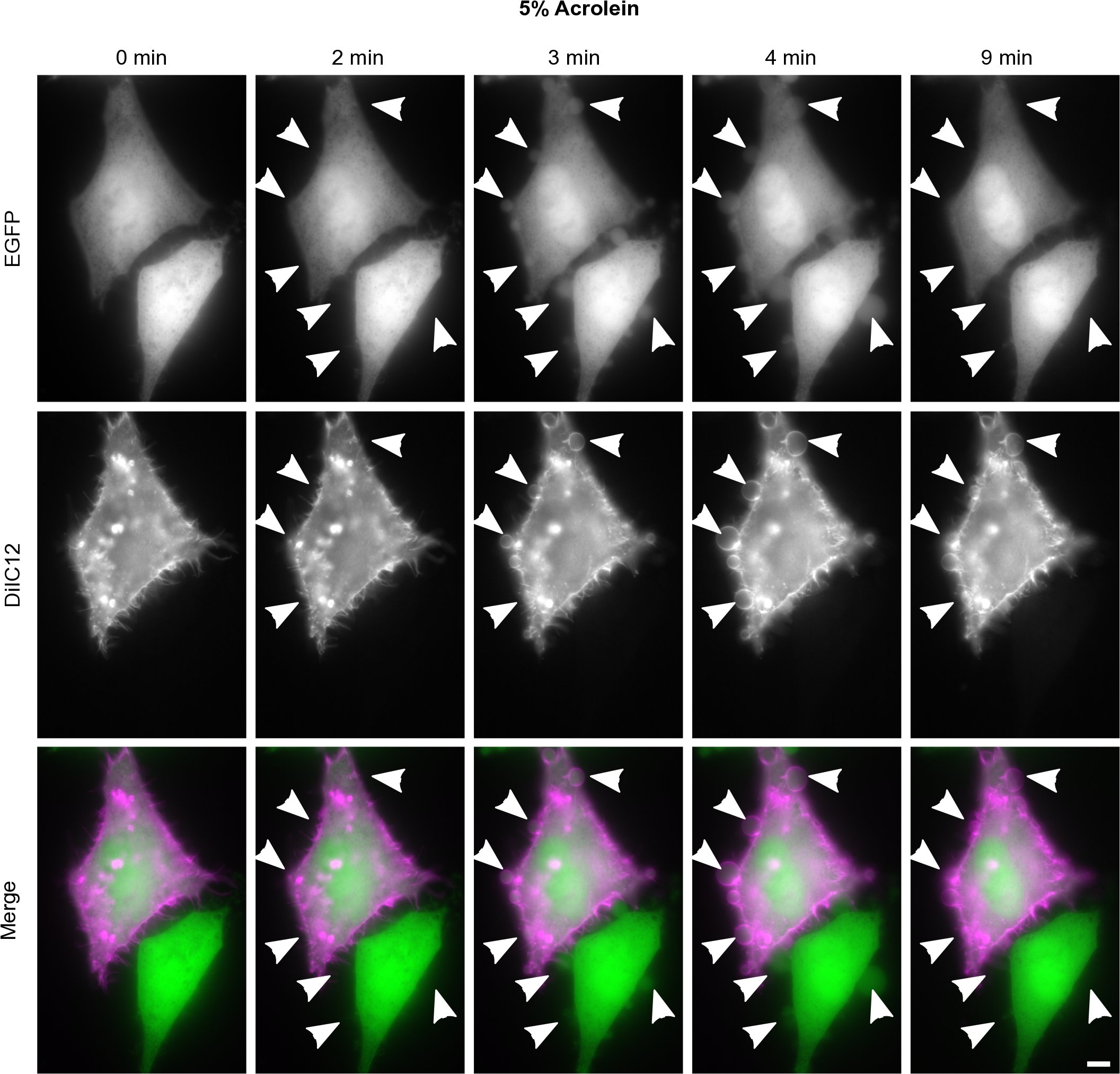


**Supplementary figure 6: Fluorescence imaging of cytosolic EGFP and the membrane-marker DiIC12 in HeLa cells during fixation with 5% acrolein**

Shown are representative fluorescence microscopy images of HeLa cells transfected with cytoplasmic EGFP (upper row, green in lower row) and stained with the membrane marker DiIC12 (middle row, magenta in lower row) during the course of fixation in 5% acrolein. The contrast is maximized for each individual image to correct for the loss of fluorescence during fixation. White arrowheads point to membrane blebs filled with cytoplasmic EGFP that develop during fixation. Note that in contrast to formaldehyde fixation, EGFP fluorescence is already lost from the blebs within the first 10 min of fixation and the contrast between nucleus and cytoplasm already changes. Scale bar: 10 μm


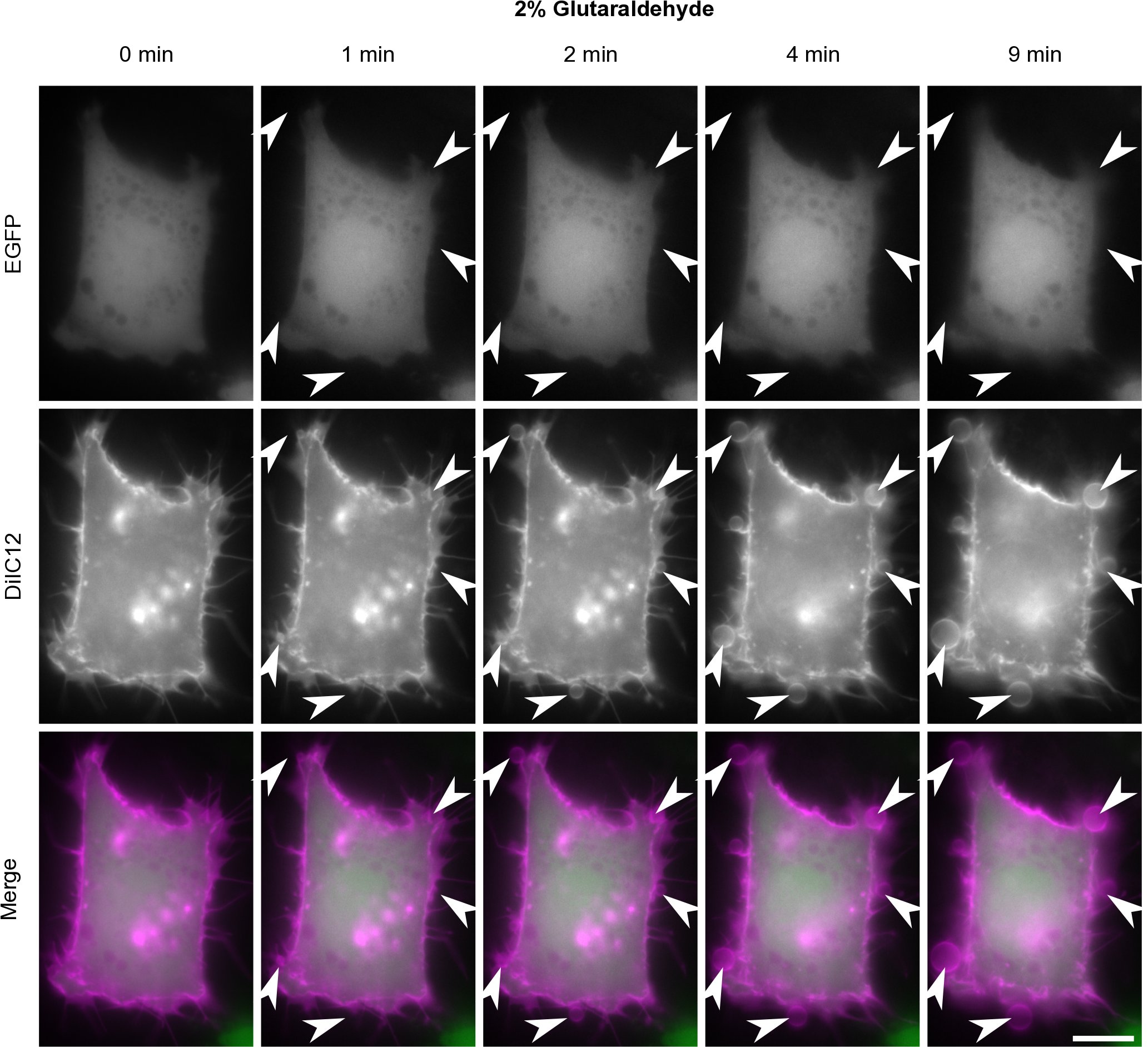


**Supplementary figure 7: Fluorescence imaging of cytosolic EGFP and the membrane-marker DiIC12 in HeLa cells during fixation with 2% glutaraldehyde**

Shown are representative (n=11 cells from 6 independent experiments) fluorescence microscopy images of HeLa cells transfected with cytoplasmic EGFP (upper row, green in lower row) and stained with the membrane marker DiIC12 (middle row, magenta in lower row) during the course of fixation in 2% glutaraldehyde. White arrowheads point to emerging plasma membrane blebs that develop during fixation and are devoid of cytoplasmic EGFP (upper row). Scale bar: 10 μm

**Supplementary figure 8: Quantification of the loss of fluorescencence of cytosolic EGFP and mCherry upon fixation with aldehydes**

**A)** Shown is the fluorescence intensity of HeLa cells transfected with cytosolic EGFP and mCherry after fixation with 4% formaldehyde and 1% glutaraldehyde normalized to their fluorescence before fixation. n=23 (EGFP) and 18 (mCherry) cells from 3 independent experiments; ***: significantly different using student’s t-test p<0.0001 **B)** Shown is the fluorescence intensity of HeLa cells transfected with cytosolic EGFP 1 min (black circels) and 10 min (red squares) after fixation with 4% formaldehyde (4% FA; n=18 cells from 3 experiments) 4% formaldehyde and 1% glutaraldehyde (4% FA 1% GA; n=23 cells from 3 experiments), 2% glutaraldehyde (2% GA; n=13 cells from 3 experiments) or treated with PBS (n=12 cells from 3 experiments) as a control normalized to their fluorescence before fixation. Significance differences between the means were tested with an ordinary one-way ANOVA and a Tukey post-hoc test. Note: The significant increase in fluorescence between 1 and 20 min after fixation with 2 % GA might is in agreement with the increase in autofluorescence induced by this fixative (compare Fig 1C), whereas the loss of fluorescence using 4 % FA is in agreement with the loss of cytosolic protein through blebbing (Fig. 2 and Fig. S5). All fluorescence intensities have been normalized to the value of the same cell before application of the fixatives or PBS.

**Supplementary figure 9: Propidium iodide staining of HeLa cells during fixation**

**A)** Shown is the quantification fluorescence intensity of HeLa cells that were fixed with the indicated mixtures of formaldehyde (FA) and glutaraldehyde (GA). The cells were treated with these fixatives containing 5 μM propidium iodide at 0 min. After 30 min, cells were completely permeabilized with 20 % ethanol containing 5 μM propidium iodide. Single cells are shown as gray lines; mean +/- s.d. are shown as black lines; orange lines are mean +/- s.d. of cells that were not fixed but incubated with 5 μM propidium iodide. n=37 (2% GA, 4%FA+2%GA) or 43 (4%FA+1%GA) cells from 3 independent experiments **B)** Representative image of propidium iodide fluorescence during fixation with 4 % FA and 1% GA.


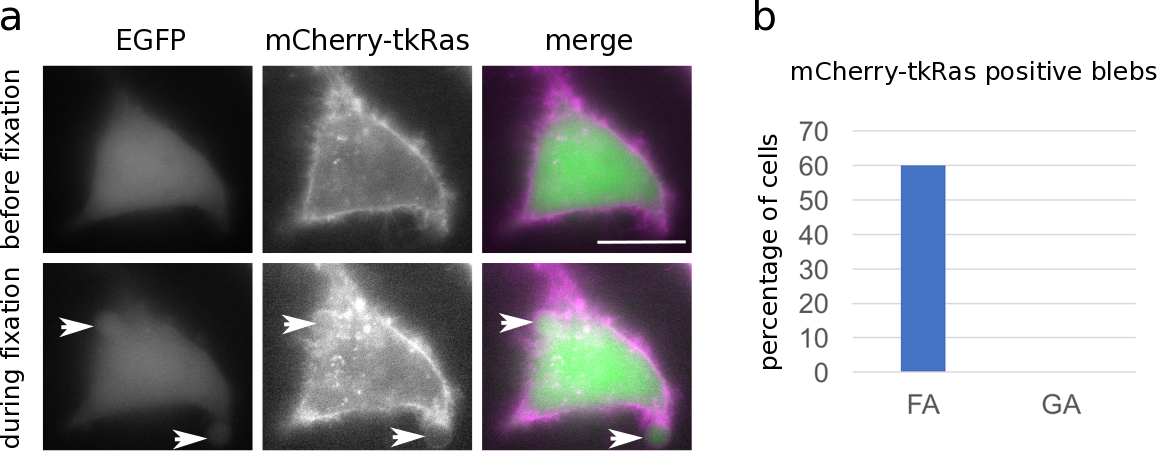


**Supplementary figure 10: Fluorescence imaging of HeLa cells expressing plasma membrane associated mCherry during fixation**

a) Shown are representative images of a HeLa cells co-transfected with cytosolic EGFP (left colum, green in right columne) and mCherry fused to the membrane anchor tkRas (last 20 amino acids of kRas; middle column, magenta in right column) before (upper row) and during (lower row) fixation with 4% formaldehyde. Arrowheads point to emerging blebs that contain mCherry-tkRas and EGFP; scale bar: 10 μm. b) Statistics of cells containing blebs positive for mCherry tkRas during fixation with 4% formaldehyde (FA; n=15 cells from 5 experiments) and 2% glutaraldehyde (GA; n=9 cells from 4 experiments). During the fixation with 2% glutaraldehyde no blebs were identified.

**Supplementary figure 11: Fluorescence imaging of cytoplasmic mCherry, mCherry bound to the plasma membran­­e and the plasma membrane marker DiIC12 in HeLa cells during fixation with 3% glyoxal and 20% ethanol at pH 4**

Shown are representative fluorescence images of HeLa cells transfected with cytoplasmic mCherry (upper row), tkRas-mCherry (middle row) or labeled with DiIC12 (lower row) upon treatment with 3% glyoxal in 20% ethanol at pH 4. Shown are images before treatment (left column), 1 and 3 min after treatment (middle columns) and after 20 min of treatment and subsequent washout with DMEM containing HEPES without phenol red (right column). Each experiment was repeated 3 times (with 8-14 cells in total) with freshly prepared solutions. Arrowheads point to emerging plasma membrane blebs. Scale Bar: 10 μm

**
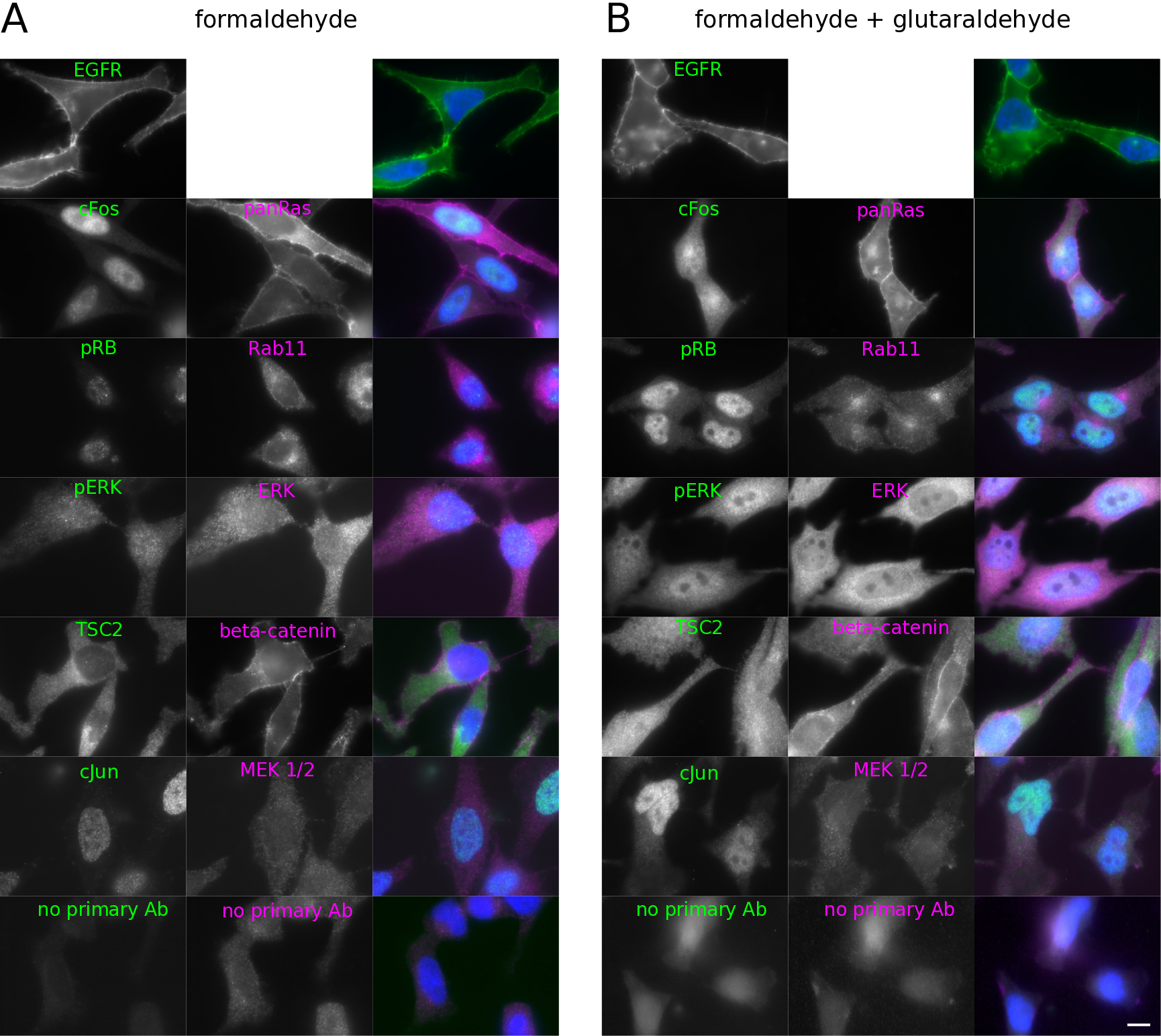
Supplementary Figure 12: Different immunofluorescence stainings in cells fixed with formaldehyde (FA) and a combination of glutaraldehyde (GA) and FA.**

HeLa cells have been fixed with 4% formaldehyde (A) or 1% GA in 4% FA (B). The cells have been permeabelised and the proteins indicated within the images in the left and the middle column have been fluorescently labelled. The contrast has been maximized in all figures individually to ensure visibility of structures. The figures in the right column show merged images of the left column (green), the middle column (magenta) and images of the dye Hoechst 33342 (blue). All experiments have been repeated three times and the images shown are representative. Antibody dilutions have been freshly prepared for each repetition and experiments after both fixations have been performed in parallel with the same antibody dilutions. Scale bar: 10 μm

**Supplementary Figure 13: Confocal and STED microscopy of alpha-tubulin labelled in HeLa cells fixed with formaldehyde (FA) and a combination of glutaraldehyde (GA) and FA.**

HeLa cells have been fixed with 4% formaldehyde (FA; left column) or 1% GA in 4% FA (FA/GA; right column). The cells have been permeabilised and labelled against alpha-tubulin by indirect immunofluorescence with the fluorescent dye Alexa-488. They have been imaged in confocal mode (upper row; apparent microtubule thickness ≈240 nm) and by STED microscopy (Iower row; apparent microtubule thickness ≈120 nm). Scale bars: 1 μm
